# Exploring HPV vaccination policy and payer strategies for opportunities to improve uptake in safety-net settings

**DOI:** 10.3389/fpubh.2023.1099552

**Published:** 2023-05-04

**Authors:** Kylie Sloan, Michelle Shin, Lawrence A. Palinkas, Shawna V. Hudson, Benjamin F. Crabtree, Joel C. Cantor, Jennifer Tsui

**Affiliations:** ^1^Department of Population and Public Health Sciences, Keck School of Medicine, University of Southern California, Los Angeles, CA, United States; ^2^Department of Children, Youth, and Families, Suzanne Dworak-Peck School of Social Work University of Southern California, Los Angeles, CA, United States; ^3^Department of Family Medicine and Community Health, Robert Wood Johnson Medical School, Rutgers, The State University of New Jersey, New Brunswick, NJ, United States; ^4^Center for State Health Policy, Rutgers, The State University of New Jersey, New Brunswick, NJ, United States

**Keywords:** HPV vaccination, health policy, safety-net, Practice Change Model, qualitative

## Abstract

**Introduction:**

We explored priorities and perspectives on health policy and payer strategies for improving HPV vaccination rates in safety-net settings in the United States.

**Methods:**

We conducted qualitative interviews with policy and payer representatives in the greater Los Angeles region and state of New Jersey between December 2020 and January 2022. Practice Change Model domains guided data collection, thematic analysis, and interpretation.

**Results:**

Five themes emerged from interviews with 11 policy and 8 payer participants, including: (1) payer representatives not prioritizing HPV vaccination specifically in incentive-driven clinic metrics; (2) policy representatives noting region-specific HPV vaccine policy options; (3) inconsistent motivation across policy/payer groups to improve HPV vaccination; (4) targeting of HPV vaccination in quality improvement initiatives suggested across policy/payer groups; and (5) COVID-19 pandemic viewed as both barrier and opportunity for HPV vaccination improvement across policy/payer groups.

**Discussion:**

Our findings indicate opportunities for incorporating policy and payer perspectives into HPV vaccine improvement processes. We identified a need to translate effective policy and payer strategies, such as pay-for-performance programs, to improve HPV vaccination within safety-net settings. COVID-19 vaccination strategies and community efforts create potential policy windows for expanding HPV vaccine awareness and access.

## 1. Introduction

Human papillomavirus (HPV) vaccination rates are lower than target levels for adolescents in the United States (US) despite a safe, effective HPV vaccine and national guidelines from the Advisory Committee on Immunization Practices recommending adolescents get fully vaccinated by age 11 or 12, and starting as early as age 9 (1). The American Academy of Pediatrics emphasizes starting HPV vaccination at age 9 and completing the 2-dose series by age 12 for reasons including, but not limited to, resulting in a more robust immune response when vaccinating at younger ages (2). To date, only about half (54.5%) of adolescents have received recommended doses of the HPV vaccine in the US, far short of the Healthy People 2030 goal of 80% (3). Urgent action is also required internationally, given the global strategy of the World Health Organization to eliminate cervical cancer, including the goal of 90% of girls by age 15 to be fully vaccinated against HPV (4). Therefore, increasing HPV vaccination continues to be a national and global priority (5, 6).

Addressing inequities in low HPV vaccine uptake communities where HPV-associated cancer burden is high continues to be paramount (7), especially as uptake of other adolescent vaccines exceeds HPV vaccine uptake, further signaling the need for targeted efforts within primary care safety-net settings (8). However, few studies to date have focused on policy and payer (e.g., health plans) strategies to increase HPV vaccination within safety-net settings. Policy strategies include both “big P” policies at the federal and state government levels, and “little p” policies which pertain to organizational or health system-level policies (9). Payer strategies, such as pay-for-performance (P4P) programs are “little p” policies involving targeted financial incentives paid to medical providers as a way to improve provider performance on quality metrics in clinics. While past research has explored HPV vaccination interactions between medical providers, patients, and families (10–12), research on HPV vaccination has focused less on perspectives of payers and policy representatives regarding potential and existing strategies to increase uptake.

Several factors at the community and policy levels influence HPV vaccination (13). Previous research on interventions to increase HPV vaccination at the policy level included factors of health insurance, state legislation, vaccine requirements, and vaccine availability (14). While the introduction of the HPV vaccine in 2006 led to proposed HPV vaccine-related legislation (e.g., school entry mandates) in multiple states, policymaking efforts since have not successfully converged to meaningfully promote HPV vaccine uptake (15). Sexuality and gender politics at the time of introduction mitigated policymaking efforts and contributed to controversy surrounding the HPV vaccine (16). More current research in the US suggests policy measures, including school entry requirements or mandates (e.g., “big P” policies), but these strategies continue to be underutilized (9). As of 2020, only five states/jurisdictions mandate HPV vaccination to attend schools (17). While mandates historically were successfully employed to reduce disease burden across the US for Tetanus, Diphtheria, Acellular Pertussis (Tdap) vaccine (required for school entry in all states) (18), and Meningococcal Conjugate Vaccine (MCV4) (required for school entry in some but not all states) (19), these policy strategies have not been as successful for HPV; thus, HPV vaccination policy strategies remain less explored beyond school entry mandates (20–22). Additional “big P” policies related to HPV vaccination include minor consent laws at the state level in which adolescents can consent to HPV vaccination without parental consent (23), state legislation regarding religious exemptions to immunizations required for school entry (24), and state-funded family planning programs like Family PACT in California, which cover reproductive health care services for residents with low incomes, such as cervical cancer screening (25).

Increasing health system focus on population health and prevention metrics, as well as innovations in community vaccination programs due to the COVID-19 pandemic, indicate that there are other potentially important opportunities for policy and payer strategies (e.g., “little p” policies) that can contribute to HPV vaccine improvement, which are largely underexplored. Prior research on payer strategies has focused on increasing access to HPV vaccines through the Vaccines for Children (VFC) program (which uses federal funds to provide vaccines at no cost to eligible children) (26) as well as private insurance, but strategies mostly pertained to cost and payment for HPV vaccines (13, 22). Additionally, while the National Committee for Quality Assurance (NCQA) continues to include an adolescent immunization quality metric in the Healthcare Effectiveness Data and Information Set (HEDIS) and Centers for Medicare and Medicaid (CMS) Star ratings, known as Combination 2 (MCV4, Tdap, HPV all received by age 13) (27, 28), the impact of these metrics on HPV vaccine improvement specifically remains underexplored. Thus, we examined the perspectives of policy and payer representatives on HPV vaccination prioritization and strategies, to inform opportunities for improving HPV vaccine uptake within safety-net settings through broader system and policy level change.

## 2. Materials and methods

### 2.1. Study design

Data for this analysis were drawn from a larger study that seeks to identify feasible evidence-based strategies (EBS) to increase HPV vaccination rates within safety-net settings through implementation of EBS in two US regions: the greater Los Angeles region and the state of New Jersey (29). The larger qualitative study used a combination of one-on-one in-depth interviews and focus groups guided by the Practice Change Model (PCM) (30), to explore perspectives, experiences, and recommendations for improving HPV vaccination from multiple groups of participants internal (clinic leaders, providers, clinic staff) and external to safety-net settings (29). This analysis focuses on a subset of one-on-one interviews with two of these groups: payer and policy representatives. This study was approved as exempt by the research team's Institutional Review Board at each study site.

### 2.2. Participants

This analysis focuses on data from in-depth interviews with 11 policy and 8 payer representatives across the two target regions. Principles of saturation and sufficiency were used to determine the sample size and the process was iterative in which we assessed for thematic saturation by group throughout data collection (31, 32). All participants were purposively sampled according to their proximity, knowledge, and/or interaction with HPV or general immunization efforts, safety-net health care delivery for pediatric/adolescent populations, or population-focused cancer prevention and control. Policy participants were health-focused policy representatives who were employed, not elected or appointed, in local-level city or county offices, state-level departments, or local or state-level non-profit organizations (see Table 1), and were purposively sampled due to their role in public health or health policy implementation. Payer participants included health plan medical directors and executives who oversaw or influenced health care delivery in safety-net settings (see Table 1). All participants were recruited using snowball sampling in each region and were offered $50 gift card incentives upon completion of interviews.

**Table 1:** Summary of HPV vaccine policy and payer representatives by region (*n* =1 9).

**Participant number**	**Group**	**Region**	**Organization**
01	Policy	Los Angeles	Academic Health System
02	Policy	Los Angeles	State Immunization Registry
03	Policy	Los Angeles	State Cancer Control Coalition
04	Policy	Los Angeles	County Immunization Program
05	Policy	Los Angeles	School District Wellness Center Nonprofit
06	Policy	Los Angeles	Office of County Supervisor
07	Policy	Los Angeles	Office of City Councilmember
08	Policy	Los Angeles	State School-Based Health Non-Profit
09	Policy	New Jersey	Office of State Legislator
10	Policy	New Jersey	State Department of Health
11	Policy	New Jersey	State Department of Health
12	Payer	Los Angeles	Managed Care Organization
13	Payer	Los Angeles	County Health Plan
14	Payer	Los Angeles	Managed Care Organization
15	Payer	Los Angeles	County Health Plan
16	Payer	Los Angeles	Consulting Firm for Publicly Funded Healthcare
17	Payer	New Jersey	Managed Care Organization
18	Payer	New Jersey	Health Insurance Plan
19	Payer	New Jersey	State Medicaid Program

### 2.3. Data collection

Interviews were conducted virtually and digitally recorded via Zoom between December 2020 and January 2022, which were transcribed verbatim and de-identified by the study team (KS, MS, JT). Interviews with payer and policy representatives lasted ~30 min. Participants were asked for their perspectives on existing priorities and strategies related to HPV vaccination and opportunities for improvement within safety-net settings. Interview guides were theoretically informed by the PCM domains of “Motivation,” “Resources for Change,” “Outside Motivators,” and “Opportunities for Change,” guiding the interviews with policy and payer participants to explore both internal and external factors that impact implementation of EBS and their interrelationships (30). Interview guides were adapted for each group (policy and payer) of participants. For example, guides tailored to the policy group asked about past experiences with developing guidelines or policies around HPV vaccination, and local or state initiatives that they would like to see for improving HPV vaccination rates in their region (see Appendix 1). In guides tailored to the payer group, questions asked about their experiences with engaging providers and practices within their network around HPV vaccination, and specific changes they would like to see in how HPV vaccines are delivered within their network, including supply, reimbursement, and metrics (see Appendix 2).

### 2.4. Research team, reflexivity, and analysis

Our multidisciplinary research team (KS, MS, JT) conducted analysis of all transcripts. Team members had varying levels of training in health policy and reflected on their positionality throughout data interpretation. PCM domains guided the thematic analysis and interpretation, which was further informed by study team members (LAP, SVH, BFC, JCC). Given the complex, multilevel factors that impact promotion and delivery of HPV vaccination (33), safety-net medical providers face a multitude of reasons for why HPV vaccination does not get systematically delivered to adolescent patients. The focus of this analysis was on policy and payer participants, who were viewed as part of the setting external to safety-net clinics, in order to provide a more comprehensive picture of the external factors that influence defining incentives for delivering HPV vaccination. Analysis using the PCM domains focused on the extent to which the strategies and priorities of policy and payer representatives acted as “Outside Motivators” that could influence the “Motivation” of members in the internal clinic setting (e.g., providers, clinic leaders/staff) and “Available Resources for Change” within clinic settings, and create “Opportunities for Change” that could improve HPV vaccination within safety-net settings.

Several rounds of analysis occurred using an immersion/crystallization approach, thoroughly described elsewhere (29). All transcripts were read and summarized with initial themes highlighted and emerging codes added to a codebook based on interview guides by the study team (KS, MS, JT). All policy and payer transcripts were coded in Atlas.ti version 9 by the study team (KS, MS, JT). The team met regularly to discuss discrepancies in code usage which were resolved through discussion to come to consensus. Finally, coded policy and payer transcripts were analyzed separately with themes identified for each group. The study team (KS, MS, LAP, SVH, BFC, JCC, JT) came together to discuss the themes and examined similarities and differences across groups and regions.

## 3. Results

Results informed by the perspectives of policy and payer representatives indicate strategies that are external to clinic settings can influence internal settings for better or worse. Five themes resulting from interview data include: (1) lack of prioritization of HPV vaccination by payer representatives in incentive-driven clinic metrics, (2) region-specific policy options for HPV vaccination improvement identified by policy representatives, (3) inconsistent motivation across policy and payer representatives to improve HPV vaccination, (4) opportunities to target HPV vaccination specifically in clinic quality improvement (QI) initiatives, and (5) acknowledging the COVID-19 pandemic as a disruption to but also an opportunity for HPV vaccination improvement (see Table 2). Overall, results point to opportunities for change that can improve HPV vaccine uptake within safety-net clinic settings.

**Table 2:** Themes and supporting quotes from policy and payer participants in New Jersey and the greater Los Angeles region organized by policy and payer strategies and priorities for action.

**Policy and payer strategies defined as “big P” and “little p” policies**	**Themes and supporting quotes**	**Priorities for action**
“Little p” policies	• 1) Payer representatives were not prioritizing HPV vaccination specifically in incentive-driven clinic metrics • “*This year, in ‘2022, as we focus on it, we're [going to] have to focus on adolescent immunizations because our rate's really low…that's actually an area of interest because that measure directly can impact our [CMS] Stars score which we need to get up.” (Participant 18, NJ payer)* • “*Since we're a Medi-Cal health plan, we get a lot of regulatory oversight…I can't recall something specifically coming down from the state in terms of any new policies or processes that they're putting place to focus in on HPV.” (Participant 14, LA payer)*	• Targeting HPV vaccination separate from other adolescent immunizations in clinic quality metrics
		
“Big P” policies	2) Policy representatives noted region-specific HPV vaccine policy options in the greater LA region and state of NJ (e.g., minor consent, Family PACT) • “*…we know that in California, I guess if you are in high school, you don't need your parent's permission to get the [HPV] vaccination. So, I think that is the other reason why we [want to] empower these youth to be able to know that this is something they can do…” (Participant 03, LA policy)* • “*I would want to see there be a requirement that by a certain age, that adolescents get the HPV vaccine. Or to allow for adolescents, at 16…to make that decision for themselves…” (Participant 11, NJ policy)* • “*I think pushing the state with Family PACT reimbursement is a feasible option.” (Participant 05, LA policy)*	• In California: Empowering adolescents (age 12 and older) to consent to HPV vaccination • In California: Expanding Family PACT to cover HPV vaccination • In New Jersey: Passing minor consent law for HPV vaccination • In New Jersey: Limiting religious exemptions to vaccinations required for school
		
N/A	3) Inconsistent motivation identified across policy and payer groups to improve HPV vaccination • “…*we want to increase our HEDIS rates because these vaccinations are the right things to do for our members” (Participant 17, NJ payer)* • “*I think they're definitely supportive [of HPV vaccination]… the question was how not to burden the physicians…So, they are open to different strategies but they have to be kind of limited disruption in the workflow.” (Participant 01, LA policy)* • “*You know it's really easy for staff to put blinders on and—for example with all due respect to your study and the focus on HPV vaccine—yeah, it's important but it's only one thing that's important for adolescent health.” (Participant 13, LA payer)*	• Identifying clear HPV vaccination champions within health policy organizations and health plans
		
“Little p” policies	4) Targeting of HPV vaccination in quality improvement initiatives suggested across policy and payer groups • “*I think focus is all we need…It's medically indicated and it just requires continued focus and monitor. Whatever we measure can improve, whatever we don't measure won't improve…We've got to measure and then give that feedback back to our providers as how well they're doing.” (Participant 15, LA payer)* • “*I think incentives for prevention and for immunizations to teens would help. You know, movie ticket[s]…Amazon gift cards or stuff like that.” (Participant 16, LA payer)* • “*Now, that [quality improvement] program includes member outreach where they see that there's a gap. By extension, we know that HPV is often a gap, so they can send reminder postcards [to members].”(Participant 17, NJ payer)*	• Creating quality improvement or value-based programs specifically for HPV vaccination
N/A	5) COVID-19 pandemic viewed as both a barrier and opportunity for HPV vaccination improvement across policy and payer groups • “*The big focus now is on COVID vaccinations…it's really [going to] be COVID and influenza for the time being as being priority vaccinations. So, those are the two ones that we're focusing on right now. (Participant 14, LA payer)* • “*So, I think accessibility is a big one that also we've learned a lot about with COVID that just making sure that it's easy for the patient to go to the clinic or to go to their doctor to go get vaccinated [for HPV]…Making it more convenient, I think, would be a large—it would facilitate a lot more people to get vaccinated [for HPV].” (Participant 02, LA policy)* • “*Incorporating it [HPV] into COVID vaccine events, I would think would be helpful right now… I think incorporating it [HPV] into back-to-school events…both educating families and/or making vaccines available at school registration.” (Participant 08, LA policy)* • “*…our company has a lot of goals in a lot of different areas and we're like, ‘This is the year of health equity and social determinants of health' and all that [kind of] stuff. But the bottom line is vaccination's people's prevention, and as a healthcare insurance company that pays out claims, that's what we're all about: preventing disease.” (Participant 18, NJ payer)*	• Employing COVID-19 vaccination strategies and community efforts for HPV vaccination

### 3.1. Theme 1: few payer representatives are prioritizing HPV vaccination in incentive-driven clinic metrics

Overall, payer representatives we interviewed in both regions spoke broadly about adolescent immunizations and did not prioritize HPV vaccination specifically in QI initiatives, including P4P and value-based programs. As one participant from a health plan in LA County shared:

“*I don't believe that HPV or adolescent immunizations today are a part of our pay-for-performance [P4P] piece…The specificity for HPV and the VIP [value improvement plan]…I don't think it's one of the yet stated targets by itself. We look at those every year and decide what we're [going to] add and delete from the pay-for-performance [P4P]…” (Participant 15, LA payer)*

Based on interviews with payers in LA, the HPV vaccine was not included in P4P programs nor were combined metrics for adolescent immunizations (e.g., HEDIS Combination 2). One NJ payer discussed how the HPV vaccine was previously a separate quality metric, but was later merged with the two other adolescent immunizations (Tdap, MCV4); however, HPV vaccination rates continue to trail behind the other adolescent immunizations rates:

“*HPV is the rate-limiting, not the other immunizations…It's the HPV that's the problem…” (Participant 17, NJ payer)*

According to this NJ payer, combining HPV together with the two other adolescent vaccinations in this plan's value-based program limited specific focus on improving HPV vaccination rates within safety-net settings.

### 3.2. Theme 2: policy representatives indicate region-specific policy options for HPV vaccination improvement

Policy representatives discussed different policy options for HPV specific to their region (greater LA region or state of NJ), beyond school entry mandates, which have not been passed in either NJ or California. Most frequently, policy representatives in California referenced minor consent laws in which adolescents do not need parental consent for HPV vaccination (already in place in California and 8 other states/jurisdictions but not NJ) (23). LA policy representatives stressed the importance of directly empowering adolescents to get vaccinated, and in NJ, minor consent laws were suggested as a policy option due to infeasibility of mandates.

Another policy representative discussed limiting religious exemptions in NJ, especially given momentum of the anti-vaccine/vaccine choice movement over the last decade, which could also complement a future school entry mandate if passed.

“*And I think that part of that [anti-vaccine/vaccine choice] movement, like, it started to really get ginned up because of the flu vaccine getting passed and because of the meningitis vaccine [MCV4]. And then there was a fear that pushing the HPV vaccine would then even give them more ammunition. And so, we changed our focus to try to eliminate the religious exemption…right now, a mandate is pretty fruitless because of the religious exemption that exists in New Jersey and how it's enforced.” (Participant 09, NJ policy)*

In LA, suggestions for policy changes focused more on HPV vaccine coverage and reimbursement by Family PACT, a state-wide program offering family planning services to low-income residents, in which sexually transmitted infection screening/treatment and cervical cancer screening are already covered services, but HPV vaccination is not (25). Two LA policy representatives named this policy area as feasible to target, and explained how clinics offering reproductive health services to adolescents cannot utilize minor consent without HPV vaccines being covered by this state-wide program.

### 3.3. Theme 3: inconsistent motivation across policy and payer groups to improve HPV vaccination within safety-net settings

Motivation regarding the need for change to improve HPV vaccination rates varied across policy and payer representatives who occupied diverse roles in health plans and organizations focused on population health policy. While some served as HPV champions within their organizations, such as policy representatives who actively advocated for mandated vaccination among adolescents in school-based settings, others did not perceive HPV vaccination to be of higher importance than other types of preventive care. As one LA payer shared:

“*…if you were to look at all the priorities that healthcare providers have to address, I mean, HPV? Important priority. So is cervical cancer screening…So is breast cancer screening…So is postpartum depression…” (Participant 12, LA payer)*

Payers we interviewed, particularly those who were health plan executives and physicians, stressed the importance of HPV vaccination and vaccinations generally, and some payers were aware of the need to increase HPV vaccination in clinics in order to improve quality metrics (NCQA, HEDIS, CMS Star ratings) for adolescent immunizations.

However, few payers appeared to be both motivated and interested in leading efforts for HPV vaccination improvement in their current roles. No policy or payer representatives opposed HPV vaccination, most recognized as the “right thing” as one NJ payer representative (Participant 17) put it, but many did not view their current role as critical for HPV vaccination improvement within safety-net settings. Payers pointed to competing priorities for providers or viewed the role of health plans as strictly related to vaccine reimbursement. One NJ payer stated:

“*Well, we actually have a completely open policy with regards to reimbursement. And we really don't have any barriers to that…I don't know what else would be possible since there are no barriers at the moment.” (Participant 19, NJ payer)*

Overall, while policy and payer representatives we interviewed supported HPV vaccination, few representatives emerged as clear champions for HPV vaccination improvement in safety-net settings.

### 3.4. Theme 4: policy and payer representatives identified targeting HPV vaccination specifically in QI initiatives

Policy and payer representatives in both regions described current or potential targeting of HPV vaccination in QI initiatives, including using provider or member incentives. Payers discussed using provider incentives through P4P programs, as well as vaccine administration fees. For example, one payer commented how their LA County health plan paid a higher administration fee for its contracts than other health plans to incentivize providers to deliver HPV vaccinations:

“*If they get the vaccine free from the Vaccine for Children Program, of course, they can't be compensated for something they didn't pay for, but the administration fee varies… we pay the vaccine administration fee on top of the capitated payment amount. So, it further incentivizes. We don't want practices not to vaccinate people because it takes time and they don't get paid.” (Participant 13, LA payer)*

An NJ policy representative suggested pressing payer organizations to improve engagement and outreach efforts for HPV vaccination to providers and members in order to increase rates:

“*…how do we get them to help us engage their members more? So, Medicaid, MCOs [Managed Care Organizations]…how can they engage? So, can they send out the reminders? Can they do the electronic reminders to a physician when they're doing the well visit?… ‘You got to talk about HPV.' How can we incentivize them to get people to get the shot?” (Participant 10, NJ policy)*

Additionally, some payer representatives in both regions expressed a desire to use data monitoring and sharing of HPV vaccination rates at the provider or practice levels to improve rates and bring a specific focus to HPV within their organizations.

### 3.5. Theme 5: COVID-19 pandemic as both a barrier and an opportunity for HPV vaccination according to policy and payer representatives

Policy and payer representatives in both regions cited the COVID-19 pandemic as a barrier, noting missed doses as adolescents were not going to in-person appointments, especially early in the pandemic. Some policy and payer representatives also described the politicization of vaccines, as well as an active anti-vaccination/vaccine choice movement especially in NJ, as a barrier:

“*…the whole vaccinations landscape is fairly volatile right now…hopefully, it doesn't continue to be politicized which I see has happened with COVID…” (Participant 15, LA payer)*

Simultaneously, some policy and payer representatives saw potential opportunities of how COVID-19 vaccination strategies could be translated to HPV vaccination. Strategies included bringing vaccination to communities through vaccine events, mobile vaccination, and bundling with delivery of COVID-19 vaccines, in addition to adolescent engagement using social media, youth-designed campaigns, and directly reaching parents and adolescents in schools. Policy representatives, specifically those who were government officials, also expressed new opportunities to improve HPV vaccination in their role since the pandemic began:

“*I see a very active role for people in my position, for my boss [LA County Supervisor], and vaccination efforts…right now, the public health also is just raising awareness for any number of issues, HPV being one of them.” (Participant 06, LA policy)*

Additionally, there were payer representatives in both regions who expressed opportunities for HPV vaccination due to health plans shifting toward using P4P and value-based programs that could be utilized as resources to increase HPV vaccination rates, especially given a drop in rates due to pandemic disruption. Payer representatives we interviewed also indicated opportunities for HPV vaccination due to health plans bringing increased focus to health equity, highlighted by the pandemic due to health inequities by race, ethnicity, and socioeconomic status that became more apparent and further emphasized the importance of increasing utilization of preventive health care services like vaccination.

## 4. Discussion

Public health policy and payer strategies clearly have important influences on HPV vaccination rates in safety-net settings, and our findings illustrate how these perspectives can and should be taken into account for HPV vaccination improvement in the US. Policy and payer strategies have influence that can be positive or negative in terms motivating clinics and providing available resources for change within clinic settings. Two kinds of policy strategies were offered by policy and payer representatives in our study as important for improving HPV vaccination in safety-net clinics. These included region-specific policies, or “big P” policies, such as minor consent laws and limiting religious exemptions. Both groups of participants also saw potential in “little p” policies involving financial incentives, including P4P programs, that are targeted to HPV vaccination. Finally, while our interviews with payer representatives mainly focused on how payers influence HPV vaccination in safety-net clinics, some payers also mentioned the potential strategy of their health plans improving awareness within the safety-net population of the need for HPV vaccination as part of community outreach and engagement missions. Thus, engaged representatives of policy and payer perspectives are important for informing HPV vaccination improvement within safety-net settings, as few champions currently exist within these broader levels of influence. Our findings also align with international efforts which emphasize taking a value-based approach to prevention of cervical cancer through optimal vaccination uptake (34–36).

Recent literature points to success in using P4P programs to improve vaccination rates for routine pediatric and childhood immunizations (e.g., Measles, Mumps, Rubella, Varicella, Influenza) in addition to adolescent immunizations (e.g., Meningococcal, Tdap) (37), as well as evidence for use of provider incentives and higher cancer screening rates (38, 39). There has been some evidence of P4P programs for increasing utilization of preventive care services, including certain childhood immunizations, for state Medicaid managed care programs as well (40, 41). However, few studies have examined inclusion (or lack of inclusion) of HPV in P4P programs, nationally or internationally (42, 43), and how P4P programs could be implemented in order to increase HPV vaccination rates in similar ways to prior implementation that has increased uptake of other child/adolescent immunizations.

Our findings indicated there was limited mention in interviews of health plans that serve safety-net populations specifically targeting adolescent vaccination as part of their P4P programs. Payers who did mention HPV vaccination as part of P4P programs elaborated that HPV vaccination was only part of the existing HEDIS metric, which combines HPV and two other adolescent immunizations together, thereby limiting focus on specific HPV vaccination improvement benchmarks. Thus, our findings suggest existing payer and policy metrics are not enough to bring attention to HPV vaccination to improve rates within safety-net settings.

Based on our interviews with payer representatives, we found that combining three adolescent vaccines into one quality metric is ineffective. Payers described how rates for the two other adolescent immunizations (Meningococcal, Tdap) already exceed HPV vaccination rates, and policy representatives noted how both Meningococcal and Tdap are already mandated for school entry in the two states in this study (as well as throughout the US) while HPV is not. Considering school entry mandates for HPV vaccines were described as not politically feasible by our policy interview participants, and especially in light of the COVID-19 pandemic and increased anti-vaccine sentiment discussed by both payer and policy representatives, there is a need for HPV vaccination to be targeted separately from other adolescent vaccinations. The lack of a stand-alone HPV vaccination quality metric limits the ability of health plans to monitor rates of HPV vaccination specifically and track improvements in rates over time or identify potential opportunities for intervention that could improve rates. Without specific focus on the HPV vaccine, rates of HPV vaccination will likely continue to remain lower than target levels despite a safe, available HPV vaccine already shown to effectively prevent cancer.

Future system-based work in this area should focus on engagement with broader health plan representatives and the potential for targeted programs to incentivize HPV vaccination improvements in adolescent populations. Additionally, future research should examine the dynamic of interactions between providers, payers, and policy representatives and how providers can partner with payers and policy representatives to increase HPV vaccine uptake among safety-net populations. Lastly, given parental hesitancy and concern about HPV vaccination promoting sexual activity among adolescents remains a barrier (33, 44), future research should also explore the extent that policy and payer strategies (e.g., incentives, minor consent) can overcome parental hesitancy and other barriers to vaccination.

Our study has limitations. Although we purposively recruited participants from these two groups (policy and payer representatives), the purpose of the larger parent study was to discuss change to improve HPV vaccination in safety-net settings. Policy representatives could have been focused on broader community settings rather than safety-net clinic settings, and payer representatives may have been thinking of non-safety-net and privately insured populations in addition to safety-net populations. Another limitation is that our study focused on two states/regions (New Jersey and greater Los Angeles) in the US, and findings may be difficult to generalize to other states. However, this qualitative study used purposive sampling that allowed for in-depth analysis of two regions with varying policy landscapes and payer compositions, thereby offering valuable perspective on similarities and differences across the regions. Lastly, our findings are hypothesis generating but not confirming and should be viewed as such.

### 4.1. Public health implications

Our study finds that representatives of policy and healthcare payer perspectives bring important insights about the external setting of safety-net clinics that influence the priorities and actions of internal clinic members (e.g., providers, clinic leaders/staff). Currently, many representatives of policy and payer perspectives do not view HPV vaccination as high priority (with the exception of those who were already HPV champions) which in turn can make providers and clinic leaders less inclined to prioritize HPV vaccination within clinic settings. The views of policy and payer representatives on strategies to improve HPV vaccination have largely been overlooked, but are clearly important for identifying broader population and system level changes that are necessary for HPV vaccination rates to meaningfully improve in the US. We identified opportunities for change and a need to translate effective policies and payer strategies, such as P4P programs (little “p” policies) that have been used to increase utilization of other preventive health care services (other specific childhood immunizations and types of cancer screening), to HPV vaccination, as well as big “P” policies such as minor consent for vaccination, in order to increase HPV vaccine uptake within safety-net settings.

## Data availability statement

The raw data supporting the conclusions of this article will be made available by the authors, without undue reservation.

## Ethics statement

This study involving human participants was reviewed and approved by the University of Southern California Institutional Review Board and the Rutgers University Institutional Review Board. Written informed consent for participation was not required for this study in accordance with the national legislation and the institutional requirements.

## Author contributions

JT, JC, BC, SH, and LP contributed to the conception and design of the study. KS analyzed the data and wrote the first draft. MS and JT contributed to the analysis and interpretation of interview data. All authors contributed to the interpretation of data analysis, manuscript revision, read, and approved the final manuscript.
