# Supplementary material for: Exploring HPV vaccination policy and payer strategies for opportunities to improve uptake in safety-net settings
Source: Front Public Health. 2023 May 4;11:1099552. doi: 10.3389/fpubh.2023.1099552 (PMC10192548; doi:10.3389/fpubh.2023.1099552)
Supplement: Supplementary file 1 [file Data_Sheet_1.docx]

Appendix

Appendix 1. Interview Guide for Policy Participants

GENERAL OPENING

Q1. Can you tell me a few words about your current role?

Q1a. How does your role relate to HPV vaccination among adolescents?

MOTIVATION OF STAKEHOLDERS

Q2. What are some of your past experiences in developing guidelines/policies around HPV vaccination?

Q3. How do your thoughts about HPV vaccination align with your organization?

RESOURCES FOR CHANGE

Q4. Could you tell me about some strategies that are currently used in your region to focus on or improve HPV vaccination rates?

Q5. Are there specific changes you would like to see in how HPV vaccination is addressed in your region?

OUTSIDE MOTIVATORS

Q6. Please describe how you interact with other professional/advocacy organizations, state and local health departments, payers, policymakers, school districts or other entities within the [REGION] regarding HPV vaccination.

Q7. What are some outside influences that impact HPV vaccination in your region in general?

OPPORTUNITIES FOR CHANGE

Q8. What factors will facilitate the adoption of new strategies for HPV vaccination in your region?

Q9. What are some local or state initiatives you’d like to see for improving HPV vaccination rates in your region and what would be your role in this?

Appendix 2. Interview Guide for Payer Participants

GENERAL OPENING

Q1. Can you tell me a few words about your current role?

Q1a. Could you walk me through how your role or organization’s role impacts HPV vaccination?

MOTIVATION OF STAKEHOLDERS

Q2. How would you describe your organization’s role in HPV vaccination for adolescents?

Q3. How do your feelings about HPV vaccination align with your organization?

RESOURCES FOR CHANGE

Q4. What are some of your experiences engaging providers/practices within your network around HPV vaccination?

Q5. Are there specific changes you would like to see in how HPV vaccines are delivered within your network, including supply, reimbursement, metrics?

OUTSIDE MOTIVATORS

Q6. What are some outside influences that impact the focus on HPV vaccination in your organization?

OPPORTUNITIES FOR CHANGE

Q7. What factors from a payer perspective do you think will facilitate an increase in HPV vaccination among adolescents in safety-net settings?

Q8. What are some local or state initiatives you’d like to see for improving HPV vaccination rates in your region?
